# The *achaete–scute* complex contains a single gene that controls bristle development in the semi-aquatic bugs

**DOI:** 10.1098/rspb.2018.2387

**Published:** 2018-11-28

**Authors:** Cédric Finet, Amélie Decaras, David Armisén, Abderrahman Khila

**Affiliations:** Institut de Génomique Fonctionnelle de Lyon, CNRS UMR 5242, Ecole Normale Supérieure de Lyon, Université Claude Bernard Lyon 1, 46 allée d'Italie, 69364 Lyon, France

**Keywords:** achaete–scute complex, water locomotion, adaptation, bristles, water striders, evodevo

## Abstract

The semi-aquatic bugs (Heteroptera, Gerromorpha) conquered water surfaces worldwide and diversified to occupy puddles, ponds, streams, lakes, mangroves and even oceans. Critical to this lifestyle is the evolution of sets of hairs that allow these insects to maintain their body weight on the water surface and protect the animals against wetting and drowning. In addition, the legs of these insects are equipped with various grooming combs that are important for cleaning and tidying the hair layers for optimal functional efficiency. Here we show that the hairs covering the legs of water striders represent innervated bristles. Genomic and transcriptomic analyses revealed that in water striders the *achaete–scute* complex, known to control bristle development in flies, contains only the *achaete–scute homologue* (*ASH*) gene owing to the loss of the gene *asense.* Using RNA interference, we show that *ASH* plays a pivotal role in the development of both bristles and grooming combs in water striders. Our data suggest that the *ASH* locus may have contributed to the adaptation to water surface lifestyle through shaping the hydrophobic bristles that prevent water striders from wetting and allow them to exploit water surface tension.

## 1. Introduction

The semi-aquatic bugs (Hemiptera: Gerromorpha) are freshwater or marine insects that occupy various water surface niches worldwide [1–4]. These insects are thought to have derived from a terrestrial ancestor that evolved the ability to stand and generate efficient movement on the water [5]. Early-diverging lineages occupy transitional zones and walk both on land and water, whereas derived lineages evolved rowing as a novel mode of locomotion on the open-water surface [1,4]. Water surface invasion is directly associated with the ability of these insects to support their body weight on the water–air interface by exploiting surface tension [5–7]. An important adaptive morphological trait that has been critical to this transition is the evolution of hydrophobic leg hairs, whose density, morphology and orientation contribute to water repellency [6,8,9]. In addition, the complex body hair layers confer hydrophobicity to the semi-aquatic bugs and protect the animal from wetting and drowning. The body of the semi-aquatic bugs is generally covered by two hair layers [10]: a dense layer of microtrichiae (short hairs) close to the body surface is thought to play the role of waterproofing in case of submergence, whereas stiff macrotrichiae (long hairs) are thought to play the role of rainproofing [11]. The semi-aquatic bugs maintain the anti-wetting properties of the hair architecture through meticulous care using grooming combs consisting of rows of stiff hairs that are present on the distal tibiae [12]. In spite of their ecological importance, the nature of the hairs covering the leg and those forming the grooming combs remains elusive. These hairs might alternatively be innervated mechanosensory structures (bristles) or non-innervated cuticular projections (trichomes) [13].

In the fly *Drosophila*, the four genes of the *achaete–scute* family encode basic helix–loop–helix factors that are master regulators of bristle development [14,15]. *Achaete* (*ac*), *scute* (*sc*) and *lethal of scute* (*l'sc*) have redundant proneural functions and promote the formation of neural precursors, whereas *asense* (*ase*) is expressed in neuronal precursors. Loss-of-function mutant flies for *ac* and *sc* display a notum devoid of bristles [16]. Similarly, loss of *l'sc* leads to the loss of body bristles whereas ectopic expression of *l'sc* in the fly notum produces additional bristles [17]. All four genes are intronless, oriented in the same orientation and clustered in a 100 kb region containing numerously shared and interspersed cis-regulatory elements [18,19]. Genetic re-arrangements within the complex lead to mutant phenotypes owing to disruption of the *cis*-regulatory organization [20].

The ancestral *achaete-scute* complex (AS-C) in insects contained two genes: an *ase* gene and an *achaete–scute homologue* (*ASH*) gene which has undergone independent duplications in the Diptera [21]. In the flour beetle *Tribolium castaneum*, *Tc-ASH* is required to promote neural precursor formation and could play the role of multiple duplicated proneural *ac–sc* genes present in species such as *Drosophila* [22]. Moreover, the expression pattern of *Tc-ase* is highly conserved between *Drosophila* and *Tribolium* suggesting a conserved ancestral function for *asense* genes [22]. In addition to the conservation of their function, *ac–sc* genes show a conserved genomic structural organization between Diptera and Coleoptera [21]. It is widely thought that the organization of the complex and the presence of shared *cis*-regulatory regions prevent separation of the genes.

Given the key role that hydrophobic bristles play in water strider biology, we hypothesized that changes to the *ac–sc* gene family could have occurred during the evolution of the Gerromorpha lineage. To address this question, we investigated the genomic organization of *ac-sc* genes in 14 species of the Gerromorpha in comparison with other insect lineages. We generated and compiled a unique dataset of AS-C proteins across insects, including available transcriptomes and genomes of early-diverging lineages. Furthermore using RNA interference (RNAi), we examined the role of this locus in the development of hydrophobic bristles in multiple species of Gerromorpha. Our findings suggest that the gene *ase* was lost in the Gerromorpha lineage and the only remaining member of the *ac–sc* family plays a key role in the formation of various bristles in this group of insects.

## 2. Material and methods

### (a) Data collection

Phylogenetic markers were identified in available genomes or transcriptomes by tBLASTn using a set of selected *Drosophila melanogaster* genes as a probe. Species names are indicated by the following prefixes Aae: *Aedes aegypti*, Aca: *Aplysia californica*, Aga: *Anopheles gambiae*, Ame: *Apis mellifera*, Apa: *Aquarius paludum*, Bge: *Blattella germanica*, Bmo: *Bombyx mori*, Bta: *Bemisia tabaci*, Caq: *Catajapyx aquilonaris*, Cca: *Ceratitis capitata*, Cle: *Cimex lectularius*, Cpa: *Cylindrostethus palmaris*, Csa: *Cupiennus salei*, Dci: *Diaphorina citri*, Dma: *Daphnia magna*, Dme: *Drosophila melanogaster*, Dpo: *Dendroctonus ponderosae*, Dpu: *Daphnia pulex*, Eda: *Ephemera danica*, Eaf: *Eurytemora affinis*, Foc: *Frankliniella occidentalis*, Gbu: *Gerris buenoi*, Hha: *Halyomorpha halys*, Hst: *Hydrometra stagnorum*, Htu: *Husseyella turmalis*, Hvi: *Homalodisca vitripennis*, Hvu: *Hydra vulgaris*, Lde: *Leptinotarsa decemlineata*, Ldi: *Limnoporus dissortis*, Lfr: *Limnogonus franciscanus*, Lfu: *Ladona fulva*, Llu: *Limnephilus lunatus*, Lmi: *Locusta migratoria*, Mam: *Microvelia americana*, Mdo: *Musca domestica*, Mex: *Medauroidea extradentata*, Mfu: *Mesovelia furcata*, Mhe: *Metrobates hesperius*, Mlo: *Microvelia longipes*, Mmu: *Mesovelia mulsanti*, Mmus: *Mus musculus*, Nlu: *Nilaparvata lugens*, Nvi: *Nasonia vitripennis*, Oci: *Orchesella cincta*, Ocu: *Oiovelia cunucunumana*, Ofa: *Oncopeltus fasciatus*, Pac: *Paravelia conata*, Pap: *Pyrrhocoris apterus*, Pba: *Pogonomyrmex barbatus*, Pbr: *Platyvelia brachialis*, Pbu: *Paravelia bullialata*, Pdu: *Platynereis dumerilii*, Phu: *Pediculus humanus*, Pve: *Pachypsylla venusta*, Ran: *Rhagovelia antilleana*, Rob: *Rhagovelia obesa*, Rpr: *Rhodnius prolixus*, Rze: *Rhagoletis zephyria*, Sma: *Strigamia maritima*, Smi: *Stegodyphus mimosarum*, Sst: *Stridulivelia strigosa*, Ste: *Stridulivelia tersa*, Tca: *Tribolium castaneum*, Tlo: *Triops longicaudatus*, Xla: *Xenopus laevis*, Zne: *Zootermopsis nevadensis*. The novel sequences generated for this analysis have been deposited in the EMBL database with specific accession numbers (electronic supplementary material, table S1).

### (b) Phylogenetic analysis

Nucleotide sequences were aligned with MUSCLE [23], manually adjusted and selected blocks were used for phylogenetic reconstruction. Maximum-likelihood (ML) searches were performed using RAxML v.8 [24] under the site-homogeneous LG+*Γ* model. One hundred bootstrap replicates were conducted for support estimation. Bayesian phylogenetic analyses were performed using MrBayes 3.2 [25] under the GTR+*Γ* model. We ran two chains for at least 1 000 000 generations and removed the first 250 000 generations as burn-in. The different nucleotide sequence alignments and tree files are available from the Dryad Digital Repository at: http://dx.doi.org/10.5061/dryad.rc454pc [26].

### (c) Ancestral state reconstruction

Ancestral reconstruction of the number of grooming combs on the different legs was performed using R software. ML methods were adapted to discrete characters (ace, package ape [27]) and the package phytools [28] was used for generating graphical representations. The simplest model ‘ER’, with equal transition rates across all categories, was the best both with Akaike information criterion and likelihood comparisons.

### (d) Microscopy

For transmission electron microscopy (TEM), adult tarsi were transversally cut into several pieces and fixed in 2% glutaraldehyde in 75 mM sodium cacodylate buffer (pH = 7.3) overnight at 4°C. Samples were washed in cacodylate buffer and post-fixed in 1% osmium tetroxide for 1 h. Samples were then dehydrated in ethanol and embedded in epoxy resin. Samples were sectioned on a Leica UC7 ultramicrotome, stained with uranyl acetate and lead citrate and imaged with a Philips CM120 TEM at 80 kV. For scanning electron microscopy (SEM), adult bugs were fixed in 4% paraformaldehyde : heptane (ratio 1 : 3) for 20 min at room temperature. Samples were examined by using a Hitachi S800 SEM.

### (e) *In situ* hybridization

Dissected embryos were fixed in 4% paraformaldehyde : heptane (ratio 1 : 3) for 20 min at room temperature, washed several times in cold methanol and then rehydrated through a methanol series to phosphate buffered saline-Tween 20 0.05%. Embryos were prehybridized for an hour at 60°C in hybridization buffer (for composition, see [29]) prior to addition of digoxigenin (DIG)-labelled RNA probe overnight at 60 °C. Blocking step was performed in 1% bovine serum albumin prior to incubation with anti-DIG antibody coupled with alkaline phosphatase for 2 h at room temperature. Embryos were washed several times before revelation with nitro-blue tetrazolium chloride/5-bromo-4-chloro-3′-indolyphosphate in alkaline phosphatase buffer. Embryos were mounted on slides in Hoyer's medium and photographed on a Zeiss Axio observer microscope. The polymerase chain reaction primers we used in this study are listed in the electronic supplementary material, table S2.

### (f) Parental RNAi

Gene knockdown of *ASH* using parental RNAi was conducted following the protocol described in [29]. To obtain stronger phenotypes, *Mesovelia mulsanti* embryos were placed at 30–31°C which allowed them to develop faster. Technically, this method shortens the delay between the injection of the double-stranded RNA and the late expression of *ASH* transcripts.

## 3. Results and discussion

### (a) Nature and structure of leg hairs

**Figure 1.**
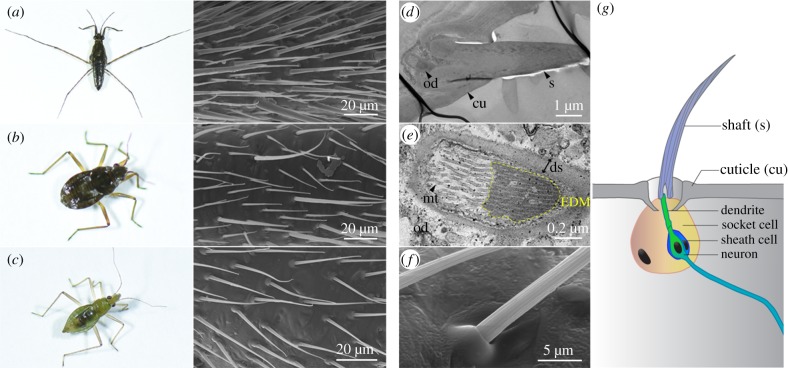
Hydrophobic leg hairs are bristles. Three Gerromorpha species have been investigated: *Gerris buenoi* (*a*), *Microvelia americana* (*b*) and *Mesovelia mulsanti* (*c–f*). All species have hydrophobic bristles on legs that are clearly visible using SEM (*a–c*). In *Me. mulsanti*, high magnification pictures show the socket cell at the base of the bristle, and the ridges of the bristle resulting from the inner actin organization (*d–f*). In *Me. mulsanti*, TEM pictures show that bristles are innervated structures with an outer dendritic segment underneath each single bristle (*d,e*). (*g*) Schematic representation of a bristle and associated structures. cu: cuticle, ds: dendritic sheath, EDM: electron-dense materials, od: outer dendritic segment, mt: microtubule, s: shaft.

The legs of water striders are covered by layers of hairs of different sizes, which form a cushion between the leg and the water surface thus preventing the leg from breaking surface tension [6,9,10,30]. Although some authors informally refer to these structures as hairs/setae [5,6,8,9], bristle-like setae [31] or bristles [32], their cellular origin is unknown. In the water strider *Gerris buenoi* (Gerridae), leg tarsi are covered with at least two types of ‘hairs’ based on their size and that are regularly arranged along the leg (figure 1*a*). This feature is shared by the microveliid *Microvelia americana* (figure 1*b*) but not the mesoveliid *Me. mulsanti* where only one ‘hair’ type can be detected (figure 1*c*). Differences in the density of hairs are also observed across species (figure 1*a–c*). SEM images show a cuticle protuberance at the base of each hair (figure 1*f*), reminiscent of the socket cell of innervated bristles in insects [33] (figure 1*g*). TEM imaging on leg hairs revealed the presence of an outer dendritic segment underneath every single hair (figure 1*d,e,g* and electronic supplementary material, S1). We therefore conclude that the numerous structures found on Gerromorpha legs are innervated bristles.

### (b) Diversification of grooming combs in Gerromorpha

Grooming, or active cleaning of body parts with specialized grooming structures, is a critical activity in the life of insects [34]. Grooming allows care of the body surface through the removal of contaminants [35,36], parasitoids [37] and pathogens [38]. Grooming also facilitates the distribution of hydrocarbons and antiseptic secretions on the body surface [39,40], as well as displacement behaviour in stressful conditions [41]. In Gerromorpha, grooming combs are of critical importance as they are used to keep the hair layers tidily arranged to prevent the leg from breaking water surface tension and the bug from drowning [5,12] (electronic supplementary material, videos S1–S3).

**Figure 2.**
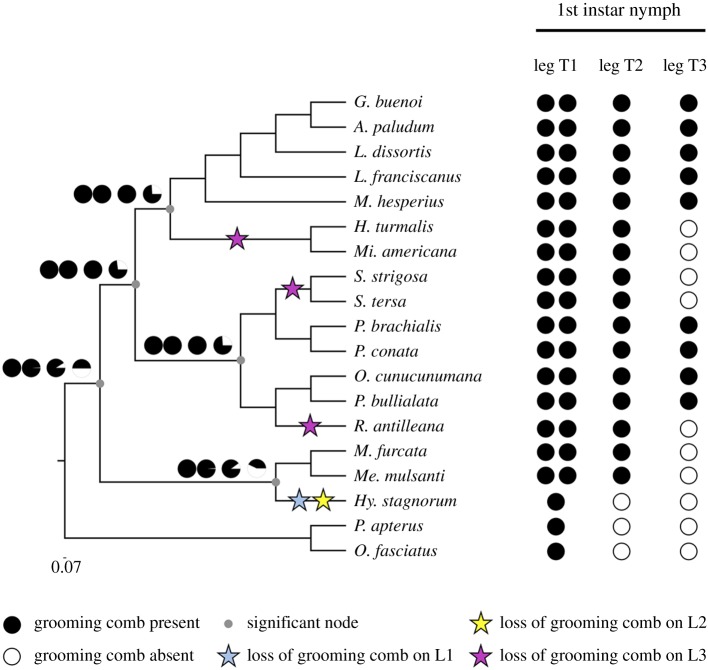
Evolutionary history of grooming combs in Gerromorpha. Presence/absence of grooming combs are mapped onto a phylogenetic tree of representative species. The phylogenetic trees, obtained through maximum-likelihood and Bayesian reconstructions, were conducted under the LG + *Γ* and the GTR + *Γ* model, respectively. Support values are shown in the electronic supplementary material, figure S2.

We found tibial grooming combs to be present in all semi-aquatic bugs we have investigated in this study. However, the number of grooming combs varies between legs in a given individual, as well as across species (figure 2). First instar nymphs have two grooming combs on the foreleg and one grooming comb in midleg tibiae in all Gerromorpha, except in *Hydrometra stagnorum* which has one grooming comb in the foreleg and none in the midleg. The reconstruction of the plesiomorphic state predicts that the common ancestor of Gerromorpha had two grooming combs in the forelegs and a single one in the midlegs. This result suggests that the loss of one of the two grooming combs of the forelegs and that of the midlegs occurred in the lineage leading to *Hy. stagnorum* (figure 2). This species is known to preferentially live on solid substrates [4], which might explain the loss of grooming combs.

The presence/absence pattern of the hindleg grooming comb is more labile across the species we investigated. The eight Gerromorpha species that lack grooming combs on the hindleg are not clustered in the phylogenetic tree, which argues for independent events during the course of evolution. However, statistical tests did not support either of the two possible hypotheses over the other: the ancestor had a hindleg grooming comb versus the ancestor lacks a hindleg grooming comb. In the event of hindleg grooming comb present in the ancestor, at least three independent losses would have occurred during the diversification of Gerromorpha (figure 2). Conversely, if the ancestor was missing hindleg combs, at least three independent gains took place in semi-aquatic bugs. According to published work in true bugs (Hemiptera: Heteroptera), the presence of tibial grooming combs on all legs is regarded as the most plesiomorphic state [42]. Moreover, the loss of grooming combs on the different legs appears to be a very common trend in terrestrial bugs [42]. It is therefore tempting to envision that the last common ancestor of extant Gerromorpha had a hindleg comb that has been independently lost in several lineages.

### (c) Conservation of *achaete-scute* complex genomic architecture

**Figure 3.**
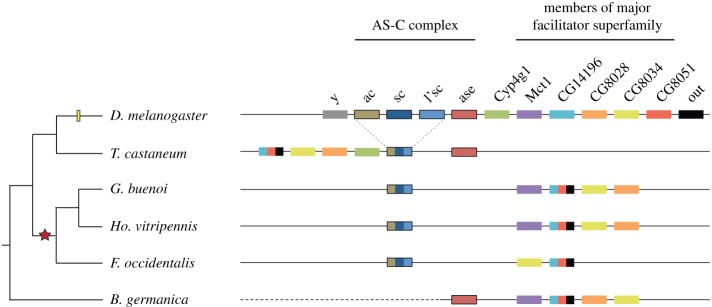
Syntenic organization of the *achaete–scute* locus in representative insects. The *D. melanogaster ac–sc* locus, which is used as a reference, is arbitrarily limited to 12 genes with specific colours. Single-copy genes depicted with colour combinations in other species mean that duplication(s) of this gene occurred in the lineage leading to the Diptera. Yellow bar: gene duplications specific to *D. melanogaster*; red star: loss of *asense*.

The structures covering the legs of the Gerromorpha being innervated bristles, it is reasonable to consider bristle specification genes as candidates to explain the specific characteristics of these leg bristles. The analysis of the AS-C gene complex is essential to understanding the contribution that proneural genes have made to the evolution of epidermal structures in insects. Whereas AS-C genes have been extensively investigated in flies, little is known about the molecular evolution of these genes across insects. To evaluate how conserved the extended genomic locus is in other insect genomes, we conducted both content and synteny analyses of this locus in two hemipterans (*G. buenoi* and *Homalodisca vitripennis*), one thysanopteran genome (*Frankliniella occidentalis*) and one blattodean genome (*Blattella germanica*). Strikingly, we failed to detect *asense* in the genome of *G. buenoi*, *Ho. vitripennis* and *F. occidentalis* species. To confirm the absence of *asense* in these genomes, we performed synteny analysis, and we took advantage of the fact that the extended genomic locus of the whole AS-C complex exhibited a set of conserved features across the Holometabola (figure 3). In the fly *D. melanogaster*, the four AS-C genes are flanked by the genes *yellow* (*y*) and *Cytochrome P450–4g1* (*Cyp4g1*) and are present in the same position in many other insects [21] (figure 3). First, the two genes *y* and *Cyp4g1* that constitute the boundary markers in the Holometabola have not been identified in the vicinity of the AS-C in the three non-holometabolan genomes we investigated (figure 3). Conversely, we identified several other markers, especially major facilitator superfamily genes, whose homologues are present within the AS-C locus in most insect species (figure 3). Second, we showed that genes which flank the position of the missing *asense* gene (*ASH* in the 5′ region; major facilitator superfamily genes in the 3′ region) are conserved across insects. This result suggests that *asense* was lost in the lineage leading to the Hemiptera (*G. buenoi*, *Ho. vitripennis*) and the Thysanoptera (*F. occidentalis*), and this loss is not associated with a reshuffling of the surrounding genomic locus.

### (d) Evolution of the *achaete–scute* complex in the Hemiptera

**Figure 4.**
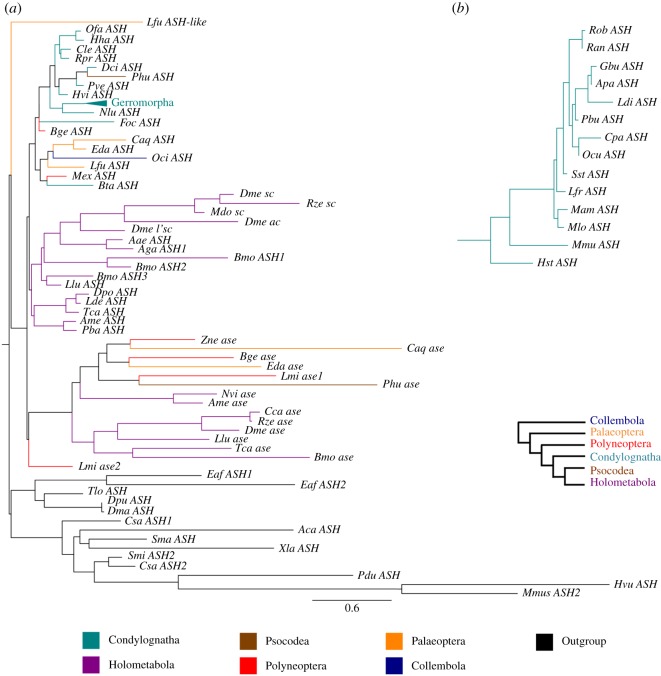
Phylogram of the 77 taxon analyses obtained through maximum-likelihood and Bayesian reconstructions were conducted using LG + *Γ* and GTR + *Γ* models, respectively. (*a*) Full tree depicting all insect taxa used in this analysis. (*b*) Zoom on Gerromorpha clade. Support values are shown in the electronic supplementary material, figure S3. Scale bar indicates a number of changes per site.

To further confirm this lineage-specific loss of *asense*, we searched for putative orthologues of *asense* in a broader sampling among insects. Using publicly available insect sequence datasets [43,44] along with in-house generated transcriptomes of 14 species of the Gerromorpha, we assembled sequence data of 77 AS-C genes and performed ML and Bayesian phylogenetic reconstruction. This sequence-based phylogenetic reconstruction identified both orthologues of *ASH* and *asense* genes in most insect lineages (figure 4, electronic supplementary material, table S3). Furthermore, both *ASH* and *asense* clades include sequences from early-diverging lineages of insects and hexapods but exclude non-hexapod sequences. Non-hexapod genes grouping closely to both *ASH* and *asense* clades are probable pro-orthologues of the *ASH* and *asense* duplicated genes. We conclude with confidence that the last common ancestor of the extant insects possessed at least two AS-C genes, corresponding to the precursors of the *ASH* and *asense* clades. This transcriptome analysis, together with the characterization of the AS-C genomic locus, suggests that the *asense* gene has been lost in the lineage leading to both Hemiptera (true bugs) and Thysanoptera (thrips) (Condylognatha). Both of these groups have sucking mouthparts and their monophyletic relationship was recently confirmed [45]. This finding represents, to our knowledge, the first reported case of *asense* gene loss in insects.

### (e) Expression of *achaete-scute homologue* in developing Gerromorpha embryos

**Figure 5.**
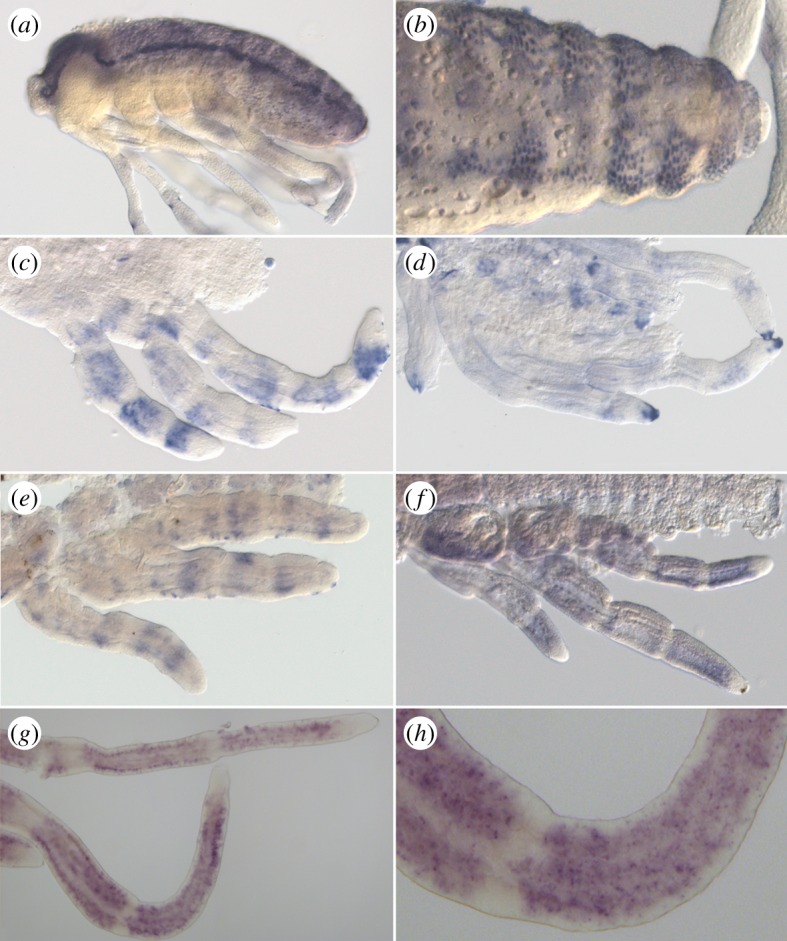
*In situ* hybridization showing *ASH* expression pattern. (*a,b*) Staining of *ASH* in *Mesovelia mulsanti*. *ASH* is expressed during central nervous system development in ectodermal cell clusters (*a*) that are progressively restricted to neural precursors (*b*). (*c,d*) Staining of *ASH* in *Microvelia americana*. In post-katatrepsis embryos, *ASH* is expressed in discrete large transverse bands along the leg (*c*). This staining is restricted to the distal tip of the tarsus in later stages (*d*). Staining of *ASH* in *Gerris buenoi* (*e–h*). Similarly to *Mi. americana* embryos, *ASH* expression pattern is dynamic in legs in sharp transverse bands (*e*), in larger bands (*f*), then in spotty domains (*g,h*).

To determine the role of the unique AS-C gene during development in the Gerromorpha, we investigated the expression of *ASH* by *in situ* hybridization in *G. buenoi*, *Me. mulsanti* and *Mi. americana*. At mid-embryogenesis, *Mmu-ASH* is expressed in ectodermal cell clusters throughout the central nervous system (CNS), especially in the head and abdominal segments (figure 5*a,b*). These cell clusters are likely to correspond to the presumptive neural precursors, suggesting that *Mmu-ASH* acts as a proneural gene in *Me. mulsanti*. Because we also detected CNS expression for *Mam-ASH* and *Gbu-ASH* (data not shown), it is reasonable to conclude that *ASH* has a proneural function in all Gerromorpha. These expression domains mirror those of *ac-sc* genes known in Coleoptera [22], Diptera [46,47] and Lepidoptera [48].

We also detected *ASH* expression in transverse stripes in all legs in post-katatrepsis embryos. In *G. buenoi*, the stripes are first sharp and narrow (figure 5*e*) before the domain of expression expands throughout leg segments (figure 5*f*). The expression of *Gbu-ASH* eventually becomes restricted to numerous dots in the legs (figure 5*g,h*), each dot likely to prefigure the position of a future bristle. This expression pattern recapitulates only the early, but not the late, expression of *ac* and *sc* found in *D. melanogaster* legs [49]. In *D. melanogaster*, the predominant expression from 3 to 5 h after puparium formation is in discontinuous transverse stripes that encircle the tarsal segments. At approximately 5 h *ac* and *sc* expression in longitudinal stripes begins to appear in conjunction with the transverse stripes, and by 6 h the longitudinal stripes in each segment replace the transverse stripes [49]. We have not detected any expression in longitudinal stripes either in *G. buenoi*, *Me. mulsanti* or *Mi. americana*.

In late embryos, the expression of *ASH* is restricted to discrete regions such as the distal tip of the tarsus (*Mam-ASH*, figure 5*d*) that prefigures the location of the future claw.

### (f) *Achaete-scute homologue* gene regulates neural precursors in the Gerromorpha

**Figure 6.**
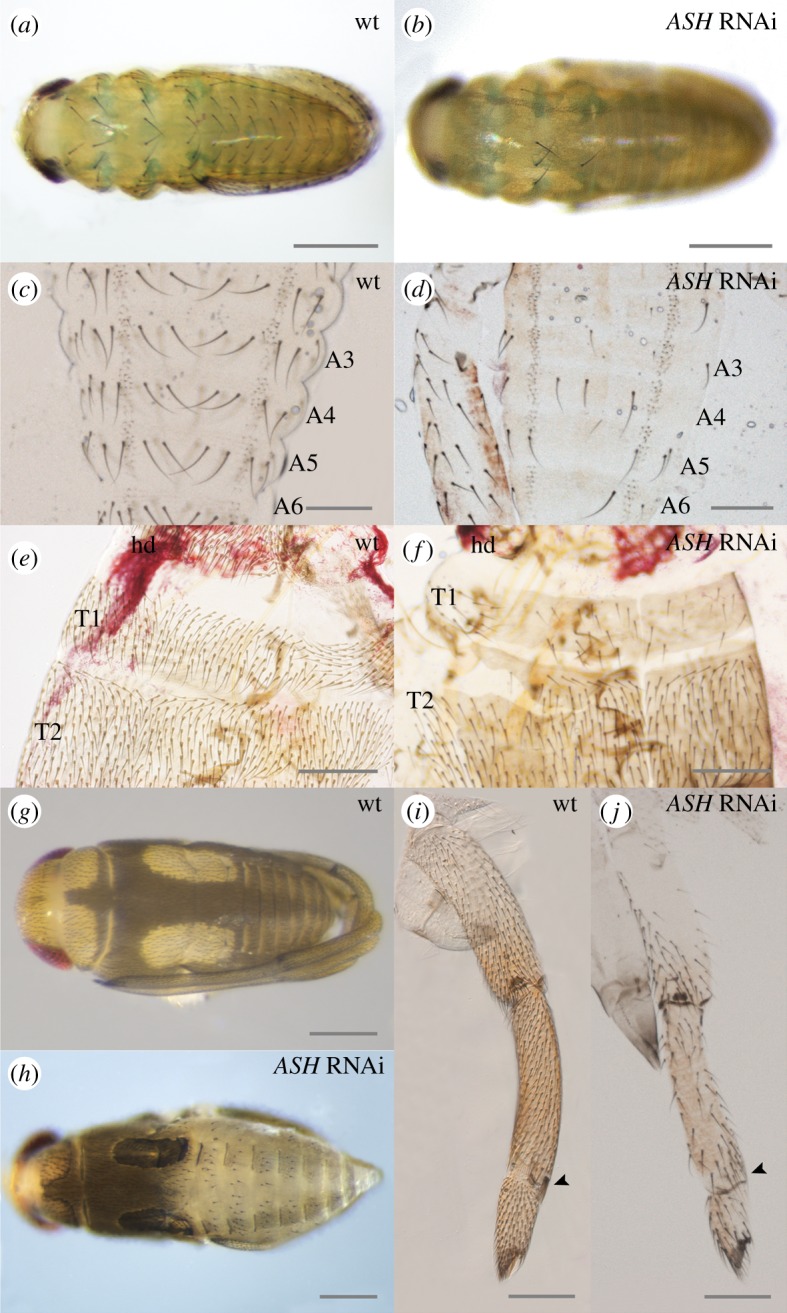
Phenotypes observed in *ASH* RNAi individuals in Gerromorpha. (*a–d*) Malformation of bristles after *ASH* RNAi in *Mesovelia mulsanti*. (*e,f*) Malformation of thoracic bristles after *ASH* RNAi in *Microvelia americana*. (*g–j*) Malformation of bristles, grooming combs, and claws after *ASH* RNAi in *Gerris buenoi*. Arrowheads show the location of grooming combs; A: abdominal segment; hd: head, T: thoracic segment. The scale bar indicates 200 m.

We used RNAi to deplete ASH function in the embryos. In *M. mulsanti*, we observed altered bristle development in 82% of *ASH* RNAi-treated embryos (*n* = 32 out of 39), ranging from a weak to a dramatic reduction in the number of bristles in the thorax and the abdomen (figure 6*a–d*), as well as in the legs (data not shown) in late embryos. The variability we observe in the knockdown phenotype is a consequence of the RNAi technique as reported in previous studies in the Gerromorpha [29,50,51]. In *Mi. americana*, only mild alterations of bristle development have been obtained (40% of embryos affected, *n* = 12 out of 30), showing missing bristles in the first two thoracic segments (figure 6*e,f*). In *G. buenoi*, we observed an altered phenotype in 97% of *ASH* RNAi-treated embryos (*n* = 208 out of 214), consisting of reduced bristle number on the whole body (figure 6*g–j*). *ASH* depletion, even subtle, consistently affected abdominal bristles (figure 6*g,h*), thoracic and head bristles secondarily and leg bristles (figure 6*i,j*). The variability in RNAi leg phenotype also ranges from mild to strong (electronic supplementary material, figure S4). The bristles that are missing or altered are not consistently the same ones between RNAi-treated embryos. However, all bristles can be affected independently of their location. It is reasonable to hypothesize that *ASH* controls the specification of all leg bristles and that the variability we see results from the partial efficiency of knockdown methods to silence the *ASH* gene.

Another important role of *ASH,* as revealed by RNAi, is the control of grooming comb development in all legs. Whereas wild-type *G. buenoi* embryos have two grooming combs on the forelegs, and one comb on mid and hindlegs (figure 2), *ASH* RNAi-treated embryos are devoid of grooming combs altogether (figure 6*j* and electronic supplementary material, figure S4). Previous studies have shown that the knockdown of the gene *Ultrabithorax (Ubx)* does not affect grooming combs on any of the legs in the Gerridae [50,52]. However, *Ubx* RNAi leads to the development of an ectopic hindleg comb in *Microvelia* and *Mesovelia* both of which lack hindleg combs otherwise [50]. This suggests that the absence of hindleg grooming combs in some Gerromorpha is owing to *ASH* repression by *Ubx*. Further experiments would be required to test this possible interaction between *Ubx* and *ASH* genes.

*ASH* RNAi-treated embryos also lack claws (figure 6*j*). This phenotype is observed for the first time in the context of an altered *ac–sc* complex. In *Drosophila*, mutation of one or several genes of the *ac–sc* complex does not affect the tarsal claw. However, the proneural gene *amos*, whose postembryonic expression prefigures the anlage of the innervated tarsal claw, is thought to be involved in the formation of sensory organ precursors in the tarsal claw [53]. Previous studies have shown that *amos* prevents bristle formation through the repression of *scute* function in *Drosophila* [54]. As a future direction, it might be interesting to test whether the genes *ASH* and *amos* act in the same developmental network during tarsal claw development in Gerromorpha.

## 4. Conclusion

The evolution of a higher density of hydrophobic leg hairs accompanied the invasion of water surface by semi-aquatic bugs. Variation in the density of bristles and the number of grooming combs found among the Gerromorpha might have evolved as an adaptation to the diversity of habitats these bugs live in [4]. We have shown these leg hairs to be bristles representing innervated structures that are known to act as mechanoreceptors. The massive number of leg bristles is associated with the semi-aquatic bugs' lifestyle that involves detection of prey trapped on the water surface, as well as detection of predators. We therefore hypothesize that the increase in leg bristle density during the course of evolution could have played a dual role: exploiting water surface tension for locomotion and developing a higher sensitivity to detect various activities through vibrations of the water–air interface including prey and predator attacks [55]. Finally, we uncovered the pivotal role of the gene *ASH* in bristle and grooming comb specification in Gerromorpha. We identified unexpected changes at the genomic *ASH* locus. Our results suggest that changes in the regulation of the gene *ASH* could have been associated with the adaptation of semi-aquatic bugs to the water surface and their subsequent diversification.

## Supplementary Material

Supplementary material

## Supplementary Material

Finet_Video1_ESM

## Supplementary Material

Finet_Video2_ESM

## Supplementary Material

Finet_Video3_ESM

## Data accessibility

Data is available from the Dryad Digital Repository at: http://dx.doi.org/10.5061/dryad.rc454pc [26].
