# Supplementary material for: The achaete–scute complex contains a single gene that controls bristle development in the semi-aquatic bugs
Source: Proc Biol Sci. 2018 Nov 28;285(1892):20182387. doi: 10.1098/rspb.2018.2387 (PMC6283939; doi:10.1098/rspb.2018.2387)
Supplement: Supplementary material [file rspb20182387supp1.pdf]

Supplementary figures and tables for :

The *achaete-scute* complex contains a single gene that controls bristle development in the semi-aquatic bugs

Authors:
Cédric Finet[1], Amélie Decaras[1], David Armisén[1], Abderrahman Khila[1]

Affiliations:
[1] : Institut de Génomique Fonctionnelle de Lyon, CNRS UMR 5242, Ecole Normale Supérieure de Lyon, Université Claude Bernard Lyon 1, 46 allée d'Italie, 69364 Lyon, France.

Corresponding authors:
abderrahman.khila@ens-lyon.fr; cedric.finet@ens-lyon.org

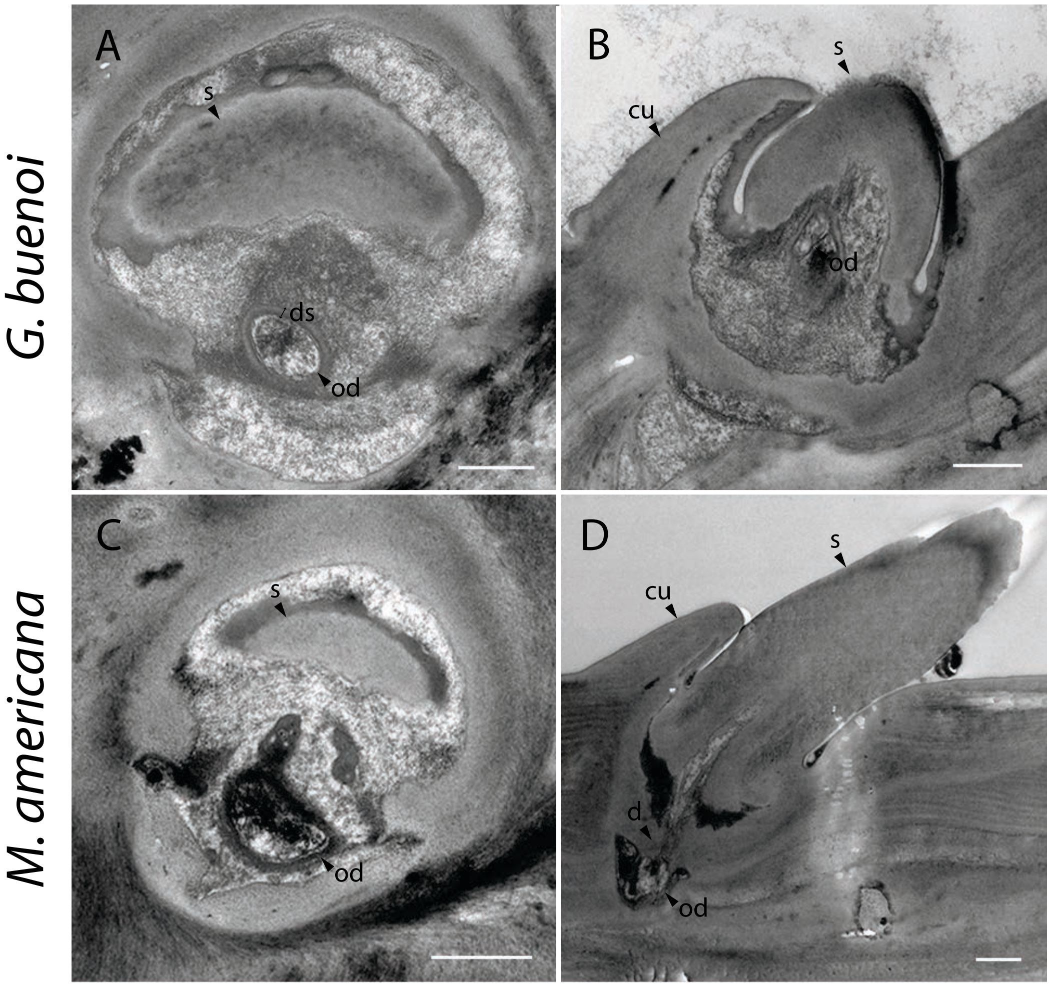

Figure S1. Ultrastructure of leg bristles in *G. buenoi* and *M. americana*. The TEM clichés show the presence of an outer dendritic segment underneath leg bristle in both *G. buenoi* (A, B) and *M. americana* (C, D). cu: cuticle, ds: dendritic sheath, oe: outer dendritic segment, s: shaft.

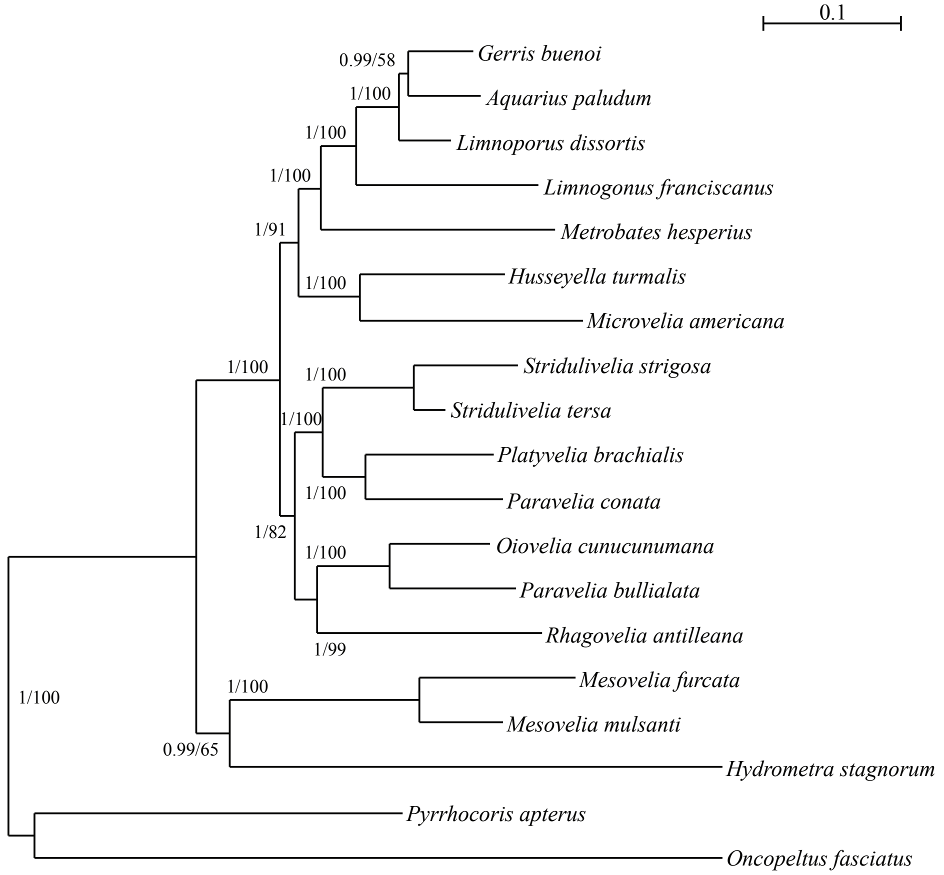

Figure S2. Phylograms of the Gerromorpha phylogeny built upon 14 markers. Support values obtained after 100 bootstrap replicates and Bayesian posterior probabilities are shown for all branches.

RAxML

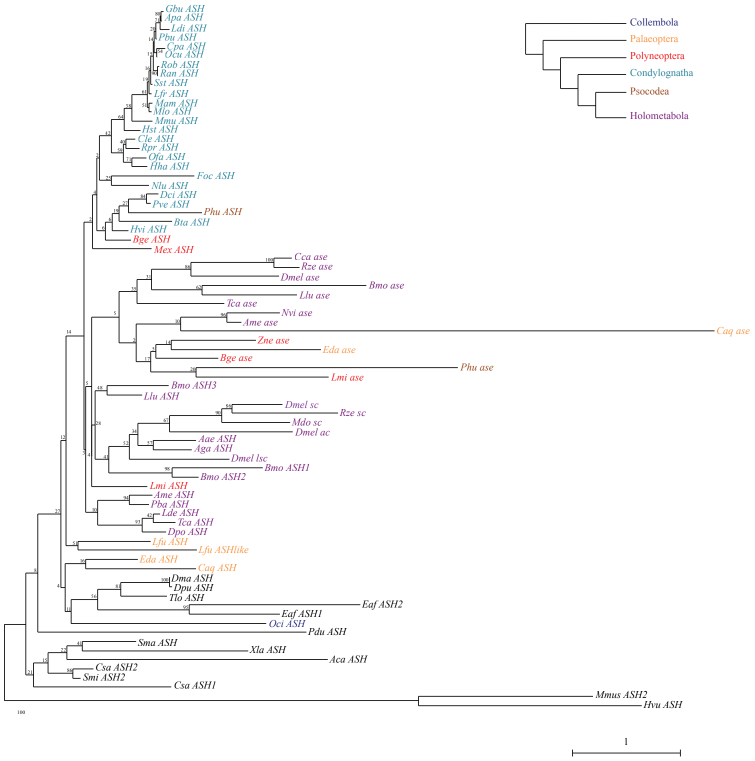

MrBayes

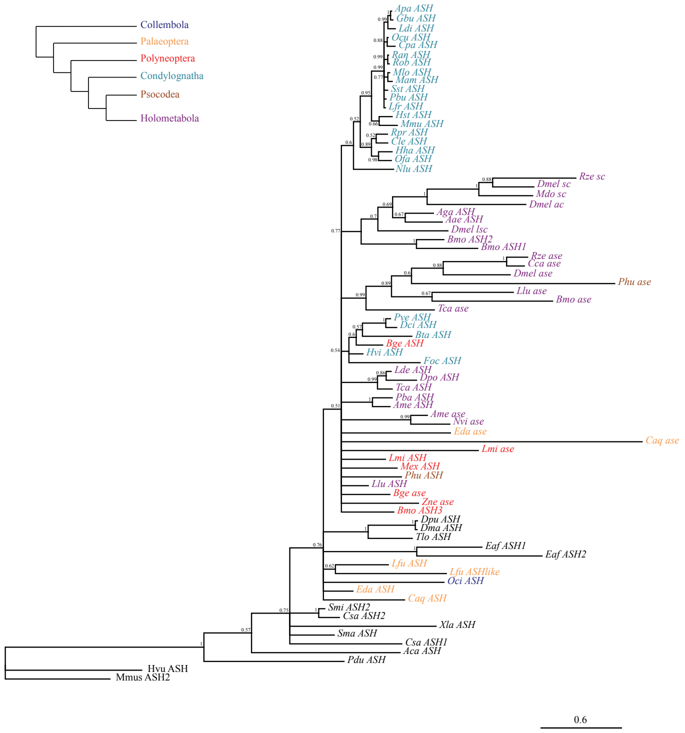

Figure S3. Phylograms of the 77-taxon analyses. RAxML Maximum-likelihood analyses and MrBayes Bayesian analyses were conducted under the LG+Γ model and the GTR+Γ model, respectively. Support values obtained after 100 bootstrap replicates and Bayesian posterior probabilities are shown for all branches. Scale bar indicates number of changes per site.

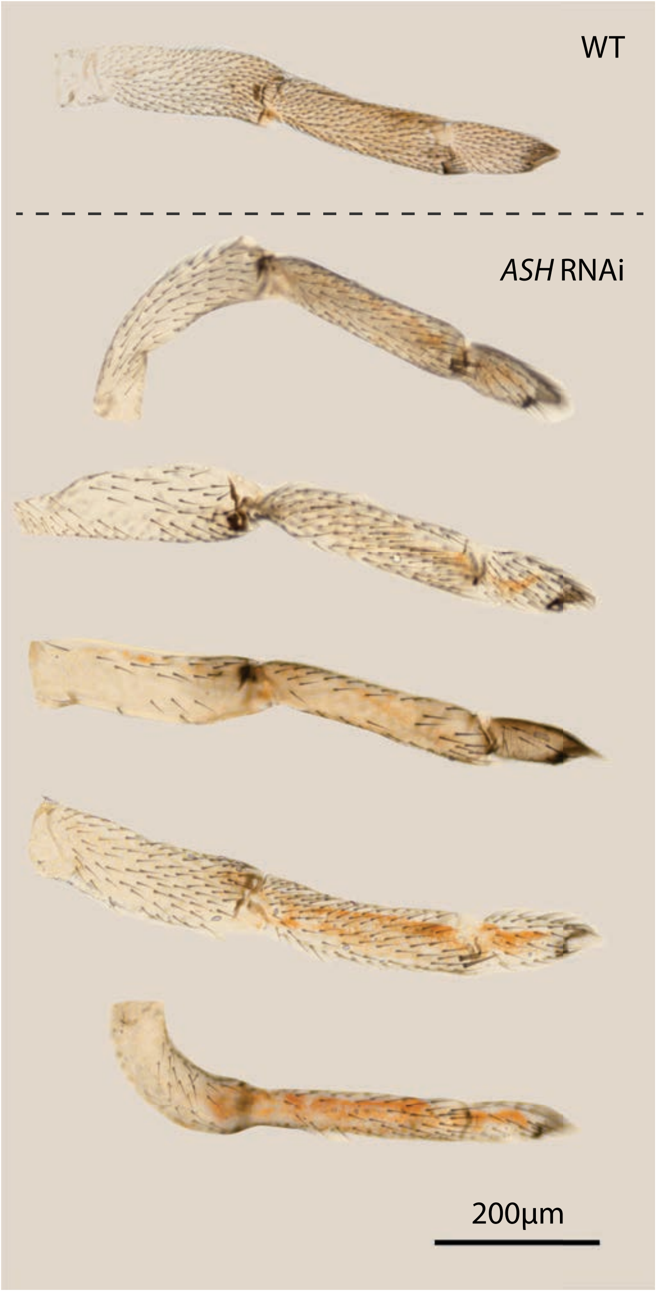

Figure S4. Variability in penetrance of *ASH* RNAi in *G. buenoi*. Top panel: wild-type leg L1; bottom panel: phenotypic variation in *dsASH* among five treated individuals.

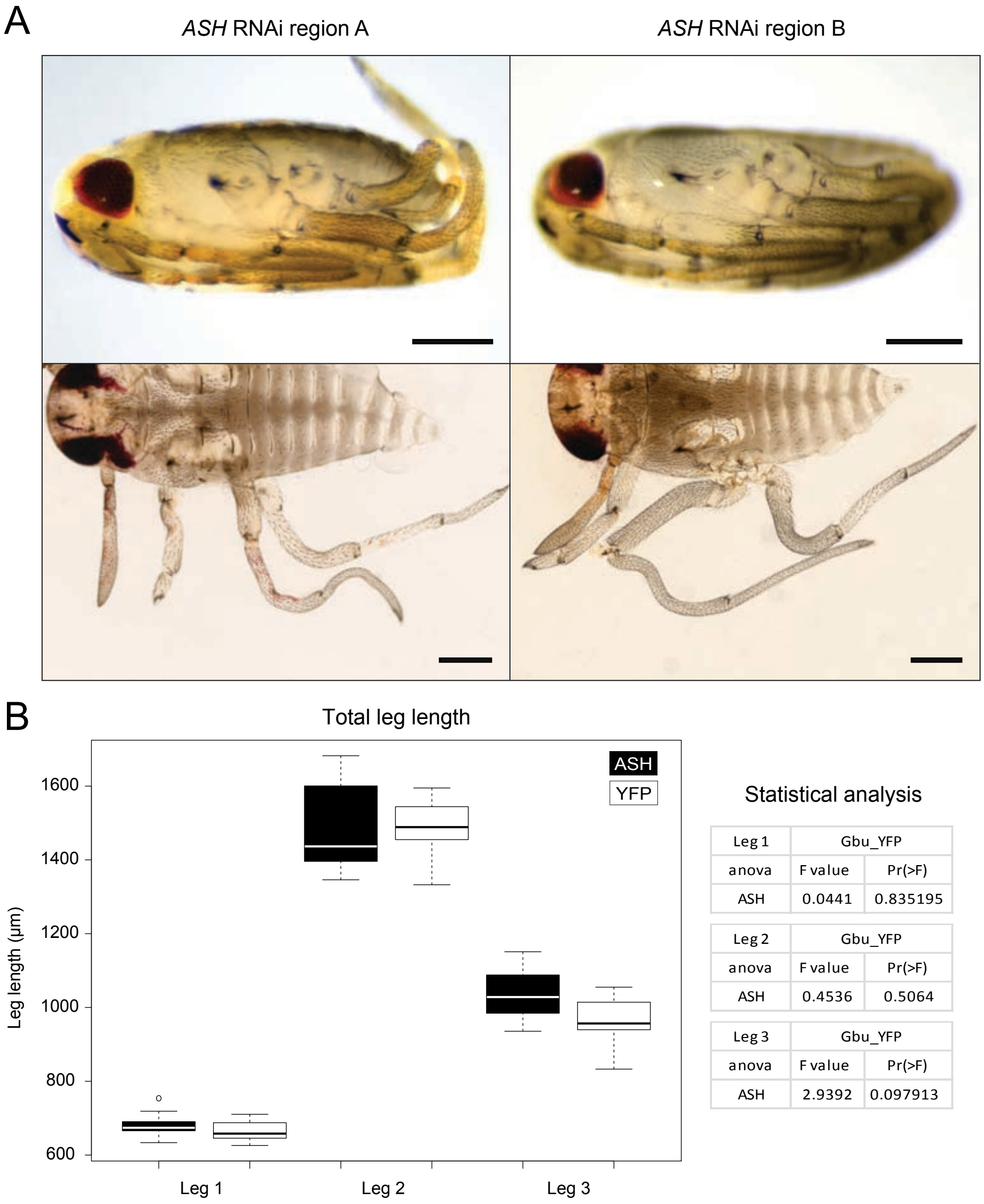

Figure S5. Phenotypic characterization of *ASH* RNAi individuals in *G. buenoi*. (A) The two non-overlapping subregions A and B targeted by RNAi show phenotypes similar to those obtained when the full *ASH* coding region is targeted. (B) The total leg length is not affected in *ASH* RNAi individuals (full construct).

Gene	Species	Accession
Antp	*Limnogonus franciscanus*	LT986725
	Metrobates hesperius	LT986723
ASH	*Aquarius paludum*	LT989878
	Cylindrostethus palmaris	LT989880
	Gerris buenoi	LT986682
	Hydrometra stagnorum	LT989889
	Limnogonus franciscanus	LT989890
	Limnoporus dissortis	LT989891
	Mesovelia mulsanti	LT986683
	Microvelia americana	LT986679
	Microvelia longipes	LT989879
	Oiovelia cunucunumana	LT989892
	Paravelia bullialata	LT989881
	Rhagovelia antilleana	LT989893
	Rhagovelia obesa	LT989882
	Stridulivelia strigosa	LT989894
COI	*Limnogonus franciscanus*	LT986734
	Metrobates hesperius	LT986735
COII	*Limnogonus franciscanus*	LT986739
	Metrobates hesperius	LT986740
COIII	*Limnogonus franciscanus*	LT989872
	Mesovelia mulsanti	LT989874
	Metrobates hesperius	LT989873
	Oncopeltus fasciatus	LT989875

Gene	Species	Accession
cyt b	*Limnogonus franciscanus*	LT986731
	Mesovelia mulsanti	LT986733
	Metrobates hesperius	LT986732
Dll	*Limnogonus franciscanus*	LT986710
	Metrobates hesperius	LT986709
GILT	*Limnogonus franciscanus*	LT986712
	Metrobates hesperius	LT986713
ND1	*Limnogonus franciscanus*	LT986741
	Mesovelia mulsanti	LT986743
	Metrobates hesperius	LT986742
Scr	*Limnogonus franciscanus*	LT986706
	Metrobates hesperius	LT986707
Ubx	*Limnogonus franciscanus*	LT986704
	Metrobates hesperius	LT986705
12S rRNA	*Limnogonus franciscanus*	LT990042
	Mesovelia mulsanti	LT990044
	Metrobates hesperius	LT990043
16S rRNA	*Limnogonus franciscanus*	LT990048
	Mesovelia mulsanti	LT990041
	Metrobates hesperius	LT990040
18S rRNA	*Limnogonus franciscanus*	LT989869
	Metrobates hesperius	LT989870
28S rRNA	*Limnogonus franciscanus*	LT989871

Table S1. Accession numbers of genes markers selected for phylogenetic analyses. *: 5,000 Insect Genome Project (i5k).

species	primer name	sequence 5'-3'
Gerris buenoi	Gbu-*ASH*-Fwd	AGCCAGAACATGGTGACTCAAAGG
	Gbu-*ASH*-Rev	TGCTGCCACCATGAGATAACGTCGA
	Gbu-ASH-Fwd1	CGAAGTTCGGCGTCCAAGAAATTG
	Gbu-*ASH*-Rev1	TGCCGTCGAAGCTCCTAAAG
	Gbu-*ASH*-Fwd-T7	**TAATACGACTCACTATAGGGAGACCAC**AGCCAGAACATGGTGACTCAAAGG
	Gbu-*ASH*-Rev-T7	**TAATACGACTCACTATAGGGAGACCAC**TGCTGCCACCATGAGATAACGTCGA
	Gbu-*ASH*-Fwd1-T7	**TAATACGACTCACTATAGGGAGACCAC**CGAAGTTCGGCGTCCAAGAAATTG
	Gbu-*ASH*-Rev1-T7	**TAATACGACTCACTATAGGGAGACCAC**TGCCGTCGAAGCTCCTAAAG
Mesovelia mulsanti	Mmu-*ASH*-Fwd	CGACGCGATGACGTCGGTTGTAGG
	Mmu-*ASH*-Rev	TGCTGCCACCACGATATAACATCAA
	Mmu-*ASH*-Fwd-T7	**TAATACGACTCACTATAGGGAGACCAC**TGCTGCCACCACGATATAACATCAA
	Mmu-*ASH*-Rev-T7	**TAATACGACTCACTATAGGGAGACCAC**CGACGCGATGACGTCGGTTGTAGG
Microvelia americana	Mam-*ASH*-Fwd	GGTCATAAAATGGTGACACAAAGA
	Mam-*ASH*-Rev	TGTTGCCACCATGATATAACATCTA
	Mam-*ASH*-Fwd-T7	**TAATACGACTCACTATAGGGAGACCAC**GGTCATAAAATGGTGACACAAAGA
	Mam-*ASH*-Rev-T7	**TAATACGACTCACTATAGGGAGACCAC**TGTTGCCACCATGATATAACATCTA

Table S2. List of PCR primers used in this study.

taxonomic group	Species name	abbreviation	ASH ortholog(s)	asense ortholog
Collembola	*Orchesella cincta*	*Oci*	1	?
Palaeoptera	*Catajapyx aquilonaris*	*Caq*	1	1
	Ephemera danica	*Eda*	1	1
	Ladona fulva	*Lfu*	1	?
Polyneoptera	*Blattella germanica*	*Bgi*	1	1
	Locusta migratoria	*Lmi*	?	2
	Medauroidea extradentata	*Mex*	1	?
	Zootermopsis nevadensis	*Zne*	?	1
Condylognatha	*Bemisia tabaci*	Bta	1	0
	Cimex lectularius	Cle	1	0
	Frankliniella occidentalis	Foc	1	0
	Halyomorpha halys	Hha	1	0
	Nilaparvata lugens	Nlu	1	0
	Oncopeltus fasciatus	Ofa	1	0
	Rhodnius prolixus	Rpr	1	0
	Aquarius paladum	*Apa*	1	0
	Cylindrostethus palmaris	*Cpa*	1	0
	Gerris buenoi	*Gbu*	1	0
	Hydrometra stagnorum	*Hst*	1	0
	Limnogonus franciscanus	*Lfr*	1	0
	Limnoporus dissortis	*Ldi*	1	0
	Mesovelia mulsanti	*Mmu*	1	0
	Microvelia americana	*Mam*	1	0
	Microvelia longipes	*Mlo*	1	0
	Oiovelia cunucunumana	*Ocu*	1	0
	Paravelia bullialata	*Pbu*	1	0
	Rhagovelia antilleana	*Ran*	1	0
	Rhagovelia obesa	*Rob*	1	0
	Stridulivelia strigosa	*Sst*	1	0
Psocodea	*Pediculus humanus*	*Phu*	1	1
Holometabola	*Aedes aegypti*	*Aae*	2	1
	Anopheles gambiae	*Aga*	1	1
	Apis mellifera	*Ame*	1	1
	Bombyx mori	*Bmo*	3	1
	Ceratitis capitata	*Cca*	2	1
	Dendroctonus ponderosae	*Dpo*	1	1
	Drosophila melanogaster	*Dme*	3	1
	Leptinotarsa decemlineata	*Lde*	1	1
	Limnephilus lunatus	*Llu*	?	1
	Musca domestica	*Mdo*	3	1
	Nasonia vitripennis	*Nvi*	1	1
	Pogonomyrmex barbatus	*Pba*	1	1
	Rhagoletis zephyria	*Rze*	3	1
	Tribolium castaneum	*Tca*	1	1

Table S3. Presence/absence of *ASH* and *asense* orthologs in the species investigated in this study.
